# Manic Symptoms during a Switch from Paliperidone ER to Paliperidone Palmitate in a Patient with Schizophrenia

**DOI:** 10.1155/2015/528370

**Published:** 2015-10-11

**Authors:** Kadir Demirci, Süleyman Keleş, Arif Demirdaş, Cafer Çağrı Korucu

**Affiliations:** Department of Psychiatry, School of Medicine, Süleyman Demirel University, 32200 Isparta, Turkey

## Abstract

Some antipsychotic drugs have treatment efficacy for mania and bipolar disorder. However, these drugs may rarely cause manic symptoms in some schizophrenic patients. We hereby report a 22-year-old female patient with schizophrenia who experienced a manic episode during a switch from paliperidone ER to paliperidone palmitate. This case is an important reminder that an abrupt switch from oral paliperidone to paliperidone palmitate may predispose certain patients to hypomanic or manic symptoms.

## 1. Introduction

Paliperidone is an antipsychotic agent which is the active metabolite of risperidone. Paliperidone palmitate, a once-monthly, long-acting injectable formulation of paliperidone, was recently marketed for the acute and maintenance treatment of schizophrenia in adults. Long-acting antipsychotics, such as paliperidone palmitate, procure continuous medication with a convenient dosing interval, aimed at enhancing treatment adherence in schizophrenic patients [1]. There are studies regarding the efficacy of paliperidone extended release (ER) in bipolar disorders [2–4]. However, there are reports describing patients with manic symptoms associated with antipsychotic treatments [5]. We found only three case reports of mania associated with paliperidone treatment in the literature [6–8].

We here report a case with schizophrenia that experienced a manic episode during a switch from oral paliperidone to paliperidone palmitate.

## 2. Case Report

A 22-year-old single woman had a 3-year history of schizophrenia. Her medical history and a review of her medical records revealed that she had been hospitalized two times because of psychotic symptoms and agitation in our hospital and treated with antipsychotic drugs, such as olanzapine and risperidone. Her treatment compliance decreased due to weight gain, galactorrhea, and akathisia. Before the most recent hospitalization, she had been undergoing treatment for her psychotic symptoms with paliperidone ER 9 mg per day, for one year. She had achieved considerable remission and had been maintaining well with good compliance for 11 months. But she had increasingly displayed paranoid delusions, auditory hallucinations, and poor treatment compliance for the last month. Hence, oral paliperidone ER 9 mg/day was discontinued, and paliperidone palmitate was started with a dose of 150 mg eq., intramuscularly to her deltoid muscle, on the day after the discontinuation of oral paliperidone. In the following week, beginning the second day after the injection, she started exhibiting increasingly deteriorating manic symptoms, including elevated mood, inflated self-esteem, and decreased need for sleep. On mental state examination, she showed pressure of speech. She reported that there was acceleration in thought and flight of ideas. She had poor insight and judgment. In addition, there was no history of substance abuse, medical or neurological disorders, depression, mania, or hypomanic symptoms. The patient did not use other psychotropics or somatic drugs when we switched from paliperidone ER to paliperidone palmitate. Physical/neurological examinations did not reveal new abnormalities. Laboratory investigations, including whole blood count, electrolytes, serum ethanol, liver, renal, and thyroid function tests, electrocardiography, and electroencephalography were unremarkable. Magnetic resonance imaging of the brain was normal. There was no family history of mental disorder. According to the Naranjo causality scale [9] (the score was 5) the adverse effect was probably due to paliperidone palmitate. On the fifth day, lithium 600 mg/day was added to her treatment, with gradual titration to 900 mg/day during the subsequent 6 days. The second dose of paliperidone palmitate was administered at 100 mg eq. as a part of the initiation regimen on the eighth day. Her manic symptoms declined at the second week after the first injection of paliperidone palmitate. Within 3 weeks she became completely euthymic. In the sixth month, the lithium treatment was gradually discontinued for a 6-week period. During the subsequent 1-year follow-up period, the patient's psychotic symptoms were managed well with paliperidone palmitate 150 mg per month and there was no reoccurrence of manic or hypomanic symptoms.

## 3. Discussion

This report presents a patient with schizophrenia who gradually developed manic symptoms after abruptly discontinuing oral paliperidone and beginning treatment with long-acting injectable paliperidone palmitate. Mania is attributed to the switching from oral paliperidone to paliperidone palmitate in this case because, based on extensive evaluations, there is no other plausible explanation. There are a few cases in the literature of mania caused by the use of paliperidone [6–8]; while in these cases the mania was induced by paliperidone, in our case mania was related to the switching from oral paliperidone to paliperidone palmitate. To the best of our knowledge, our case is the first report of mania associated with paliperidone palmitate.

It is recommended that when switching from oral antipsychotics, paliperidone palmitate should be started with a dose of 150 mg eq. on treatment day 1 and 100 mg eq. on day 8. Moreover, for patients switching from oral once-daily paliperidone tablets, treatment with paliperidone palmitate may begin 1 day after stopping oral paliperidone [1]. In our case, manic symptoms might be associated with both the discontinuation of oral paliperidone and the administration of paliperidone palmitate. Paliperidone ER has a relatively long half-life, with an elimination half-life of 23 hours after a single dosing of paliperidone [10]. In addition, *T*_max_ of paliperidone ER is 24 hr after administration; therefore, the serum concentration of paliperidone is adequate to demonstrate the efficacy at the first dosing day of paliperidone palmitate. So, the likelihood that our patient's manic symptoms were withdrawal reactions from paliperidone ER might be low.

Paliperidone, the main active metabolite of risperidone, is a new antipsychotic agent. Risperidone has antimanic effect, but it has been rarely reported to induce mania in some cases. It is suggested that risperidone-induced mania might result from blockade of D_2_ and 5-HT_2A_ receptors [11]. Similarly, paliperidone has the properties of D_2_ and 5-HT_2A_ blockading. Moreover, paliperidone has significant *α*_2_-antagonist effect and may potentially improve depression. It was probably these receptor features and antidepressant effect of paliperidone that are the cause in our patient. Evidence suggests that a single injection of long-acting paliperidone palmitate results in gradual increase in plasma level that reaches a maximum in a median *T*_max_ of 13 days and paliperidone palmitate release begins from the first day and continues for as long as 4 months [12]. Besides, by giving the two initial doses, therapeutic concentrations of paliperidone palmitate may be achieved quickly within one week and there is no recommendation for oral supplementary paliperidone during the starting regimen because of the rapid onset of therapeutic efficacy of paliperidone palmitate [12]. These pharmacological properties and probable antidepressant effects of paliperidone could have been the cause of mania in our case. The second dose of paliperidone palmitate was administered at 100 mg on the eighth day because we thought that manic symptoms could be temporary and associated with rapid antidepressant effects of high-dose paliperidone. In addition, we used lithium treatment and it was successful in treating manic symptoms induced by antipsychotic switching.

Consequently, the precise mechanisms underlying mania during the switching in this case remained unclear. This report demonstrates the importance of monitoring neuropsychiatric adverse drug reactions after the switch to long-acting paliperidone palmitate treatment in schizophrenic patients. New studies will enlighten this issue whether cross-titration strategy is a safer method than abrupt switch when switching from paliperidone ER to paliperidone palmitate or not.
